# Temporal Dynamics of Corticomuscular Coherence Reflects Alteration of the Central Mechanisms of Neural Motor Control in Post-Stroke Patients

**DOI:** 10.3389/fnhum.2021.682080

**Published:** 2021-07-23

**Authors:** Maxime Fauvet, David Gasq, Alexandre Chalard, Joseph Tisseyre, David Amarantini

**Affiliations:** ^1^ToNIC—Toulouse NeuroImaging Center, Université de Toulouse, Inserm, UPS, Toulouse, France; ^2^Department of Functional Physiological Explorations, University Hospital of Toulouse, Hôpital Rangueil, Toulouse, France; ^3^Department of Neurology, University of California, Los Angeles, Los Angeles, CA, United States; ^4^California Rehabilitation Institute, Los Angeles, CA, United States

**Keywords:** electroencephalography, electromyography, brain muscle functional connectivity, agonist and antagonist muscles, elbow extension

## Abstract

The neural control of muscular activity during a voluntary movement implies a continuous updating of a mix of afferent and efferent information. Corticomuscular coherence (CMC) is a powerful tool to explore the interactions between the motor cortex and the muscles involved in movement realization. The comparison of the temporal dynamics of CMC between healthy subjects and post-stroke patients could provide new insights into the question of how agonist and antagonist muscles are controlled related to motor performance during active voluntary movements. We recorded scalp electroencephalography activity, electromyography signals from agonist and antagonist muscles, and upper limb kinematics in eight healthy subjects and seventeen chronic post-stroke patients during twenty repeated voluntary elbow extensions and explored whether the modulation of the temporal dynamics of CMC could contribute to motor function impairment. Concomitantly with the alteration of elbow extension kinematics in post-stroke patients, dynamic CMC analysis showed a continuous CMC in both agonist and antagonist muscles during movement and highlighted that instantaneous CMC in antagonist muscles was higher for post-stroke patients compared to controls during the acceleration phase of elbow extension movement. In relation to motor control theories, our findings suggest that CMC could be involved in the online control of voluntary movement through the continuous integration of sensorimotor information. Moreover, specific alterations of CMC in antagonist muscles could reflect central command alterations of the selectivity in post-stroke patients.

## Introduction

Understanding how agonist and antagonist muscles activity is controlled during goal-directed and precise movements is an ongoing challenge in understanding neural control of human movement. In motor control and related fields, it has been demonstrated that muscular contraction is generated by an efferent motor command sent from the motor cortex to the muscles based on somatotopic organization in the central nervous system (Jasper and Penfield, 1949; Penfield, 1954). Moreover, movement control implies continuous integration of afferent and efferent information (Campfens et al., 2013) during both preparatory and online control phases of movement execution (Buneo and Andersen, 2006). This argues for the involvement of continuous communication between the brain and muscles to drive efficiently the activity of both agonist and antagonist muscles activated during the movement. The analysis of the brain’s oscillatory rhythms through event-related desynchronization (Pfurtscheller, 1992) in the beta frequency band (β, 13–30 Hz) enables characterizing increased cortical excitability during movement, thought to reflect an “activated (cortical) state with enhanced processing” (Pfurtscheller, 2001) related to motor control (Pfurtscheller and Lopes da Silva, 1999). The interactions between brain and muscles can be further analyzed through corticomuscular coherence (CMC), the use of which has been in continuous development for two decades in the fields of motor control and neuroscience. CMC can be taken as a descriptor of the brain-muscle functional connectivity, defined as a measure of the functional coupling between sensorimotor cortex and muscular activity obtained from electroencephalography (EEG) and electromyography (EMG) during muscular contraction (Conway et al., 1995; Halliday et al., 1998; Mima and Hallett, 1999; Salenius and Hari, 2003; Baker, 2007). CMC would result from the interaction between the motor cortex and contracting muscles via efferent descending motor pathways and afferent ascending somatosensory pathways (Baker, 2007; Witham et al., 2011; Campfens et al., 2013), which is consistent with evidence of the efferent and afferent components in CMC (Riddle and Baker, 2005). Even if it still remains to clearly understand the functional role of such a synchronization between the brain and muscle oscillatory signals (Bourguignon et al., 2019), most studies on CMC in motor control endorse the consensus that CMC takes part in the regulation of agonist and antagonist muscles activity (Cremoux et al., 2017; Dal Maso et al., 2017), and is related to sensorimotor integration (Baker, 2007; Witham et al., 2011). CMC is thus thought to reflect a direct regulation process occurring in the motor system via the corticospinal pathway (Conway et al., 1995; Kristeva et al., 2007).

Most of the results on the contribution of CMC to motor control have been obtained during isometric muscular contractions (Conway et al., 1995; Mima et al., 1999; Salenius and Hari, 2003). In healthy subjects, these results have highlighted that the value of significant CMC can vary according to the force level (Mima et al., 1999; Omlor et al., 2007), experimental design (von Carlowitz-Ghori et al., 2014) and the frequency band even if it occurs mainly at ∼20 Hz (i.e., in the β frequency band). The first studies analyzing CMC during dynamic contractions have shown that CMC was present during the pre- and post-movement phases, but was absent during movement (Kilner et al., 1999, 2003). However, more recent studies conducted on isokinetic (Liu et al., 2019) or cyclical (Yoshida et al., 2017) contractions have shown that the CMC magnitude is not constant over the time-period corresponding to movement, and a recent study performed on healthy subjects engaged in a squat-like task revealed that the level of CMC was different according to either the concentric, eccentric or isometric movement phases (Kenville et al., 2020). Similarly, a recent study from our group (Glories et al., 2021) showed that CMC decreased in lengthening compared to isometric contractions. Taken together, these results raise an important methodological concern regarding CMC evaluation—namely the need for a novel dynamic analysis framework to account for the time-varying changes in CMC during the time course of movement execution – and hence the need to refine and enhance our understanding of its involvement in the functional coupling between brain and muscles. This is also in agreement with the recent findings from Nijhuis et al. (2021) who highlighted dynamic modulations of CMC in healthy subjects involved in finger tapping, thus suggesting “an important role of beta band neural oscillations in […] sensorimotor synchronization.”

In line with the approach used by Chen et al. (2018) to investigate the brain mechanisms underlying the control of inter-joint synergies in post-stroke patients, the relevance of analyzing the temporal evolution of CMC is assumed to be not only fundamental, but also clinical. Indeed, previous studies have found lower CMC in stroke patients compared to healthy subjects (Mima et al., 2001; Fang et al., 2009), and have also shown that CMC peaks are more widely distributed over the scalp in post-stroke subjects (Rossiter et al., 2013). These findings may be at least partly explained by the numerous neuronal reorganizations and adaptive mechanisms that occur following stroke (Grefkes and Ward, 2014): as a consequence of the alteration of the corticospinal tract (Chollet et al., 1991) and other neuronal circuits (Ward et al., 2003), stroke implies increased activity in contralesional motor cortex or ipsilesional non-sensorimotor regions (Chollet et al., 1991; Ward et al., 2003; Gerloff, 2006) and decreased intercortical inhibitions (Grefkes et al., 2008). These impairments lead to less efficient neural drive to paretic muscles and may alter the flow of afferent and efferent information required for fine motor control, to the detriment of limb motor function and patient’s autonomy (Langhorne et al., 2011). Previous studies proposed that the remaining CMC in patients could reflect the degree of recovery after stroke (Graziadio et al., 2012; Zheng et al., 2018). The examination of temporal dynamics of CMC in stroke patients could open new insights on what extent the alteration of the neural information flow along the motor tracts contributes to motor function impairment. Referring to the perspectives offered by the analysis of the temporal evolution of corticomuscular interactions after stroke (Chen et al., 2018), a comparison of CMC dynamics during voluntary movement between healthy subjects and post-stroke patients could further help understanding the roles of CMC in motor control with potential application for the use of brain computer interfaces for rehabilitation (Tung et al., 2013).

Using a novel analysis framework, the present study compares time-varying changes in CMC during sub-movement phases of active elbow extension between control subjects and post-stroke patients, exploring possible association between alterations of motor performances and alterations of the brain-muscles communication. Such links would help understand the functional role of CMC in the control of agonist and antagonist muscles during voluntary active movements. In line both with previous results (Yoshida et al., 2017; Liu et al., 2019; Nijhuis et al., 2021) and the hypothesis of constant integration of afferent and efferent information in CMC, we first hypothesized that CMC would vary with movement time for both control subjects and post-stroke patients. We also expected to find differences in either average or instantaneous CMC parameters between control subjects and post-stroke patients, which could reflect a deficit of motor control after stroke resulting from an alteration of afferent and efferent information flows. The significance of these findings are discussed in relation to motor control theories to better understand to what extent temporal dynamics of CMC could reflect adaptive mechanisms contributing to motor performance after stroke.

## Materials and Methods

### Participants

Eight healthy control volunteers (43 ± 21 years, three females) and seventeen post-stroke patients in chronic phase (58.2 ± 12.7 years, four females), none of which was specifically trained to the task, were recruited from two different ongoing prospective studies (see Table 1 for detailed patient demographics). Patients with cognitive disorders preventing simple instruction comprehension, with an active elbow extension angle less than 20 degrees or suffering from painful movement of the paretic arm were excluded from the study. The first study, approved by the Research Ethical Committee of Toulouse University Hospitals (No. 07-0716), included five post-stroke patients and all healthy controls, and the second study, approved by Research Ethics Board (No. ID-RCB: 2017-A01616-47), included the remaining subjects. Both studies were conducted in accordance with the Declaration of Helsinki and all participants gave written informed consent.

**TABLE 1:** Participants’ demographics (M, Male; F, Female; FMA, Fugl-Meyer Assessment for upper extremity; WFMT, Wolf Function Motor Test; MCA, Middle Cerebral Artery).

Subjects	Age/Sex	Time since stroke (months)	Stroke type	Localization	FMA	WFMT
*S1*	61/M	51	Hemorrhage	Right, basal ganglia and corona radiata	38	33
*S2*	59/F	18	Ischemia	Right, cortical and subcortical territories of MCA	46	34
*S3*	69/M	19	Ischemia	Right, pons	44	46
*S4*	65/M	75	Ischemia	Right, cortical and subcortical territories of MCA	32	50
*S5*	50/M	30	Hemorrhage	Left, basal ganglia and internal capsule	42	42
*S6*	57/M	14	Ischemia	Left, posterior limb of the internal capsule	45	46
*S7*	75/M	26	Ischemia	Left, cortical and subcortical territories of MCA	26	10
*S8*	46/M	8	Ischemia	Left, cortical and subcortical territories of MCA	53	49
*S9*	65/M	116	Ischemia	Right, cortical and subcortical territories of MCA	30	36
*S10*	49/M	13	Ischemia	Right, pons	53	50
*S11*	33/F	13	Ischemia	Left, pons middle cerebral peduncles	45	48
*S12*	33/F	12	Ischemia	Left, subcortical territories of MCA	21	25
*S13*	57/M	18	Ischemia	Right, cortical and subcortical territories of MCA	41	42
*S14*	56/M	34	Ischemia	Right, cortical and subcortical territories of MCA	29	29
*S15*	75/M	12	Ischemia	Left, subcortical territories of MCA and hippocampus uncus	50	54
*S16*	74/M	34	Ischemia	Right, pons	23	28
*S17*	66/M	6	Ischemia	Left, subcortical territories of MCA	47	47
*Controls (n* = *8)*	43 ± 21/3F	–	–	–	–	–

### Experimental Procedure

The experimental procedure was the same as described in Chalard et al. (2020). Briefly, in the initial position, subjects were comfortably seated, their arms resting on a table with shoulders 80° flexed, the elbow 90° flexed and the forearm placed in front of the thorax. They were instructed to perform two series of ten full elbow extension/flexion cycles at a self-selected speed, returning to the initial position once the flexion movement has ended. Each movement cycle was preceded with an audible stimulus, with at least 10 s rest between each elbow extension and flexion. The experimental procedure was performed on both the dominant and the non-dominant arms for control subjects and on both the paretic and the non-paretic arms for post-stroke patients.

### Materials

#### Kinematics

Upper limb kinematics were recorded at 125 Hz with eight infrared cameras (model S250e, Optitrack, NaturalPoint, Corvallis, Oregon, United States). Reflective markers were placed upon breastbone, C7 vertebra, and both acromion, lateral epicondyle, ulnar styloid and second metacarpal. Arm et forearm segments were defined by acromion-lateral epicondyle and lateral epicondyle-ulnar styloid markers, respectively.

#### Electroencephalography

EEG data were continuously recorded at 1024 Hz using a 64-channel EEG cap (ActiveTwo System, Biosemi, Amsterdam, Netherlands) placed according to the international 10–20 system. Reference electrodes were included in the cap and impedance of all electrodes was maintained below 30 kΩ before the experiment start.

#### Electromyography

Surface EMG signals from triceps brachii (TB), biceps brachii (BB), brachialis (BA), and brachioradialis (BR) were recorded at 1000 Hz using disposable Ag-AgCl electrodes in bipolar configuration with an inter-electrode distance of 2 cm, by using the MP150 system with EMG100C amplifier (Biopac Systems Inc., Goleta, CA, United States). After standard skin preparation procedures (Hermens et al., 2000), the pairs of electrodes were placed over the belly of each muscle, identified from palpations and few blank tests of elbow extension, in a same manner was done in Charissou et al. (2017) for surface EMG of hand and wrist muscles. The reference electrode was placed on the right mastoid. Additionally, the EMG signals were monitored online before the experiment start to assess the good positioning of electrodes and the recordings quality.

#### Synchronization

Kinematic, EEG, and EMG data were synchronized with a common Transistor-Transistor Logic (TTL) pulse generated from the Biopac MP150 system.

### Analysis

Data preprocessing and analysis were performed offline with MATLAB 2017b (The MathWorks Inc., Natick, MA, United States). In this study, only elbow extensions of the dominant arm in controls and of the paretic arm in post-stroke patients were considered, mainly because the non-paretic arm is known to be also affected in post-stroke patients (Graziadio et al., 2012), suggesting that it may not be a suitable control for our analysis. It is noteworthy that no significant differences were found between dominant and non-dominant arms in controls.

#### Data Preprocessing

Kinematic data were low-pass filtered at 6 Hz. Elbow joint angle was calculated as the two-dimensional angle between the arm and forearm segments. Elbow angular velocity and acceleration profiles were obtained from elbow angular displacement data by finite differentiation. An angular velocity threshold over 0.01 degrees.s^–1^ was chosen to identify the beginning and end of each elbow extension movement (Chalard et al., 2020).

Continuous EMG and EEG data were 3–100 Hz band-pass and 45–55 Hz notch filtered, all filters being zero-lag fourth order Butterworth filters.

Kinematic, EMG and EEG data were segmented into epochs from −3 s prior to the beginning and + 3 s after the end of each movement.

EMG signals were visually inspected to reject epochs with movement artifacts. Additionally, for each subject, the epochs with outlier values for which EMG root mean square (RMS) was larger than twice the standard error were also rejected in order to exclude potential unexpected movements. EEG data were common average referenced and visually inspected to reject epochs with eye-blinks or face and neck muscles contractions artifacts.

The average number of remaining elbow extensions for analysis was similar between control subjects and post-stroke patients [13.9 ± 4.6 vs. 14.2 ± 3.5; *t*(20) = 0.18, *p* > 0.05].

#### Kinematic Analysis

Active elbow extension angle was calculated from the difference of the elbow joint angle between the initial and the fully extended arm positions.

As recommended by Rohrer et al. (2002), movement smoothness was quantified as the number of peaks of the acceleration profile analysis, further normalized by the mean angular velocity (de los Reyes-Guzmán et al., 2014; Chalard et al., 2020).

#### Corticomuscular Coherence Analysis

##### Corticomuscular Coherence Calculation

**FIGURE 1:**
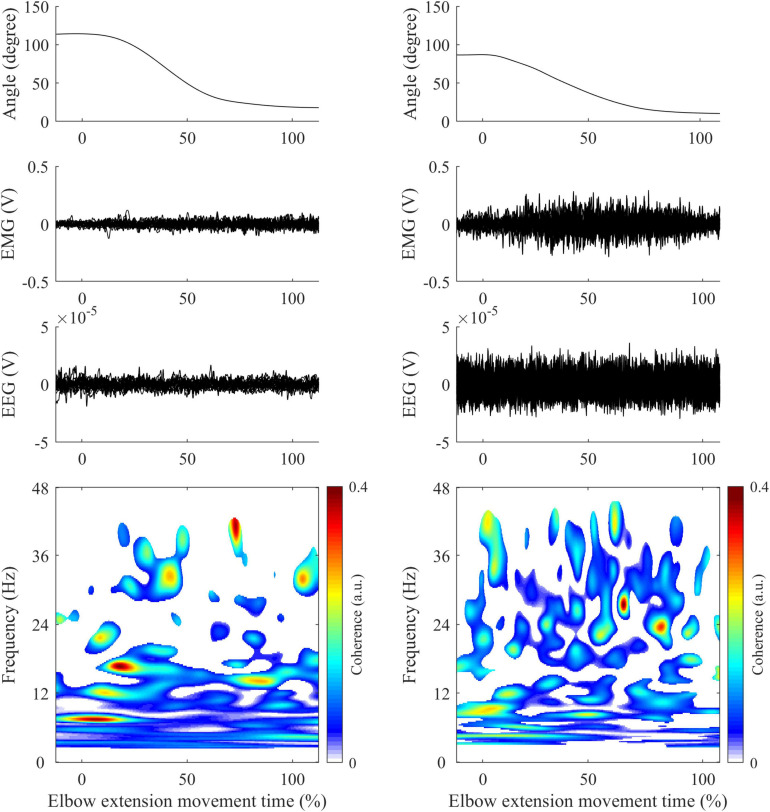
Illustration of typical recordings obtained in control (left panels) and patient (right panels) during elbow extension movement. First row: Elbow joint kinematics. Second row: Mean EMG signals from Triceps Brachii. Third row: Mean EEG signals from C3/C4 electrodes (depending on the studied arm). Fourth row: CMC computed between EMG (Triceps Brachii) and EEG signals following steps described in methods. All-time series are represented in percent of elbow extension.

CMC between EEG signals and unrectified EMG signals was first computed in the time-frequency domain using wavelet analysis with the *WaveCrossSpec* package proposed in Bigot et al. (2011) and previously used for corticomuscular coherence analysis (Cremoux et al., 2017; Dal Maso et al., 2017). In the ongoing debate on EMG rectification for coherence analysis, we clearly advocate the non-rectification of EMG signals to satisfy both theoretical arguments (Bigot et al., 2011; McClelland et al., 2012) and experimental evidence showing that EMG rectification is not suitable for coherence analysis (Ruiz-Gonzalez et al., 2019). In *WaveCrossSpec*, the wavelet parameters “nvoice,” “J1” and “wavenumber” were, respectively, set to 7, 30 and 10 to yield time-frequency transforms of full signals in the 0.002–48 Hz frequency range. These parameters set the time-frequency precision compromise to a 0.1 s – 3 Hz precision window within the β (13–30 Hz) frequency band. To cope with the issue of inter-trial duration variability that can lead to power spectrum cancelation, a normalization procedure was used to obtain EEG and EMG time-frequency power and EEG-EMG coherence spectra with time expressed as a percentage of elbow extension movement time (Fauvet et al., 2019). This normalization step is designed as to preserve frequency content of signals and enable point-wise comparison between trials of different durations. Typical recordings of kinematics, EMG from TB electrode, EEG from C3 electrode and CMC obtained in control (left) and patient (right) are presented in Figure 1.

##### Corticomuscular Coherence Detection and Quantification

In order to consider the functional reorganization of motor networks following stroke (Grefkes and Ward, 2014), the choice of interest EEG electrodes was individualized among participants. Then, for each subject, CMC was analyzed with the EEG electrode where event-related desynchronization in the β band was highest among the electrodes covering bilateral sensorimotor areas (Krauth et al., 2019) (FC1, FC3, FC5, C1, C3, C5, CP1, CP3, CP5, FCz, Cz, CPz, FC2, FC4, FC6, C2, C4, C6, CP2, CP4, CP6). The choice of the EEG electrodes showing the maximum event-related desynchronization values during movement ensures that CMC is computed from the sensorimotor regions with the maximum cortical activity. Only these selected and personalized sets of EEG electrodes were used in the following steps of analysis.

For each subject and each muscle separately, CMC was finally quantified from EEG-EMG coherence values where the interactions between the EEG and EMG was significant in the time-frequency plane with two key approaches:

• First approach: Average CMC was computed as the mean of magnitude-squared coherence values in the β band where a significant correlation between EEG and EMG was detected on the wavelet cross-spectrum (Bigot et al., 2011) over (i) the whole elbow extension movement duration, (ii) the acceleration phase of elbow extension and (iii) the deceleration phase of elbow extension. This distinction between the acceleration and deceleration phases, as indicated by the time to peak elbow angular velocity, has been made to consider the changes in the coordination and the functional role of agonist and antagonist muscles between the two phases (Chiovetto et al., 2013).
• Second approach: To investigate temporal dynamics of CMC, instantaneous CMC was computed at each time instant *t* as the mean of the magnitude-squared coherence values in the β band where a significant correlation between EEG and EMG was detected on the wavelet cross-spectrum (Bigot et al., 2011).

### Statistics

Statistical analyses were performed with Matlab built-in functions. Generalized linear models were used to test the group effect (i.e., controls vs. patients) on the mean values of active elbow extension angle, peak angular velocity, movement smoothness and average CMC computed over the entire movement and in both the acceleration and deceleration phases. Models’ results are presented as mean ± standard error (SE) difference between patients and controls, with the corresponding explained variance (R^2^) and *p*-value. The models’ quality was graphically assessed by visual evaluation of residuals normality and variance homogeneity (Anscombe, 1973). It is noteworthy that all models showed normal residuals distribution and explained at least half of the total variance. For all tests, significance was accepted at *p* < 0.05.

Inter-group instantaneous differences in angular kinematic and CMC profiles were assessed with Tmax non-parametric tests (Wilcoxon signed-rank-based) using 2000 permutations as implemented in EEGLAB, 2019 version, function *statcond* (Delorme and Makeig, 2004). As performed by Castelhano et al. (2017), *p*-values were corrected for multiple comparisons with a successive use of False Discovery Rate (Benjamini and Hochberg, 1995) and cluster-based (Maris and Oostenveld, 2007) methods.

The same statistical procedure was used to determine instantaneous differences from either the mean or the null value on CMC profiles, and to inspect the inter-group differences in the instantaneous CMC of each EEG/EMG electrode pairs. For the antagonist muscles, the periods showing significant CMC with BA, BB, and BR were pooled for this analysis.

## Results

### Elbow Angular Kinematics

**FIGURE 2:**
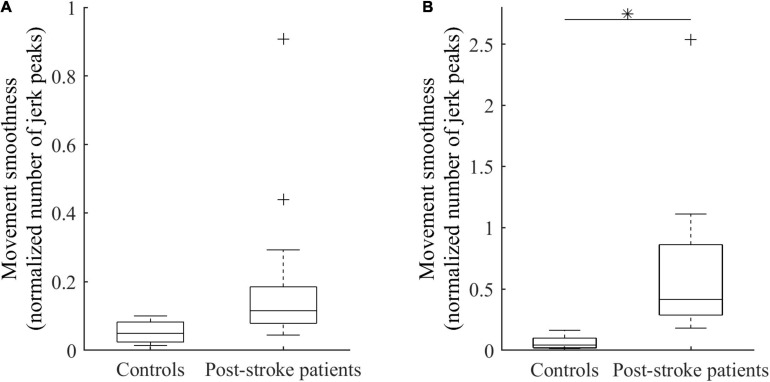
Smoothness of elbow extension for controls and patients during the acceleration phase **(A)** and the deceleration phase **(B)**. ^∗^Indicates a significant between-group difference (*p* < 0.05).

For the same functional task of elbow extension performed by both healthy controls and post-stroke patients, the analysis revealed between-groups differences in motor performance. The mean amplitude of active elbow extension was 91 ± 11 degrees for controls and 60 ± 12 degrees for patients, indicating that a significant decrease (−32 ± 2.3 degrees, *R*^2^ = 0.61, *p* < 0.01) was found in the patient group when compared to the control group. Similarly, the peak angular velocity was −0.86 ± 0.02 degrees.s^–1^ for controls and −0.58 ± 0.02 degrees.s^–1^ for patients, leading to a significant velocity peak decrease in patients (−0.28 ± 0.02 degrees.s^–1^, *R*^2^ = 0.50, *p* < 0.01). Moreover, the analysis of sub-phases of movement revealed that movement smoothness was significantly altered in patients compared with controls during the deceleration phase only (+ 0.60 ± 0.23, *R*^2^ = 0.22, *p* = 0.02; Figure 2).

### Average and Instantaneous CMC

Average CMC computed over the whole elbow extension movement duration with elbow extensor or each flexor did not significantly differ between groups (0.01 < *R*^2^ < 0.08, all *p* > 0.12). Likewise, no significant inter-group differences were observed on average CMC during either the acceleration or deceleration (0.01 < *R*^2^ < 0.06, all *p* > 0.30; and 0.01 < *R*^2^ < 0.04, all *p* > 0.35, respectively). Detailed average CMC of each group are presented in Table 2. Nevertheless, the results provided by the analysis of CMC dynamics showed differences between the two groups, revealing the substantial interest of such analyses:

**TABLE 2:** Average CMC magnitude ± SE of each group in all muscles and all movement phases.

	Full movement		Acceleration phase		Deceleration phase	
Muscle	Controls	Patients	Controls	Patients	Controls	Patients
TB	0.04 ± 0.01	0.04 ± 0.01	0.04 ± 0.02	0.03 ± 0.03	0.06 ± 0.02	0.05 ± 0.03
BA	0.03 ± 0.02	0.04 ± 0.03	0.02 ± 0.02	0.03 ± 0.03	0.07 ± 0.02	0.06 ± 0.03
BB	0.03 ± 0.02	0.04 ± 0.02	0.03 ± 0.02	0.03 ± 0.03	0.04 ± 0.02	0.05 ± 0.04
BR	0.03 ± 0.02	0.04 ± 0.03	0.02 ± 0.03	0.02 ± 0.05	0.03 ± 0.02	0.03 ± 0.04

*Statistical analyses did not reveal any inter-group differences.*

**FIGURE 3:**
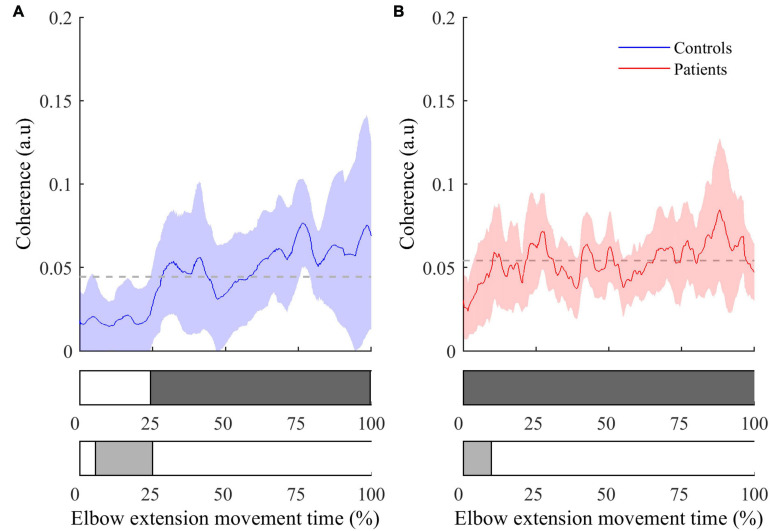
Instantaneous CMC with BA muscle (mean ± 95% CI) as a function of time expressed in percent for controls **(A)** and patients **(B)**. The colored areas inside the horizontal bars beneath the graphs indicate time periods where CMC is significantly different from mean instantaneous CMC (light gray areas) or from null value (dark gray areas). In both graphs, the dashed lines represent the average CMC computed over the whole movement duration.

• The instantaneous CMC magnitude for BA of each group is represented in Figure 3. Point-wise comparison of CMC to zero showed a biphasic pattern for healthy controls: CMC was not significantly different from zero during the first 25% of movement duration (CMC < 0.02, all *t* < 2.32, all corrected *p* > 0.05), while it was significantly above zero during the remaining time of movement (0.02 < CMC < 0.07, 2.33 < *t* < 5.91, all corrected *p* < 0.05). Conversely, instantaneous CMC remained continuously above zero during the whole movement duration for post-stroke patients (0.03 < CMC < 0.09, 2.74 < *t* < 7.30, all corrected *p* < 0.05).

**FIGURE 4:**
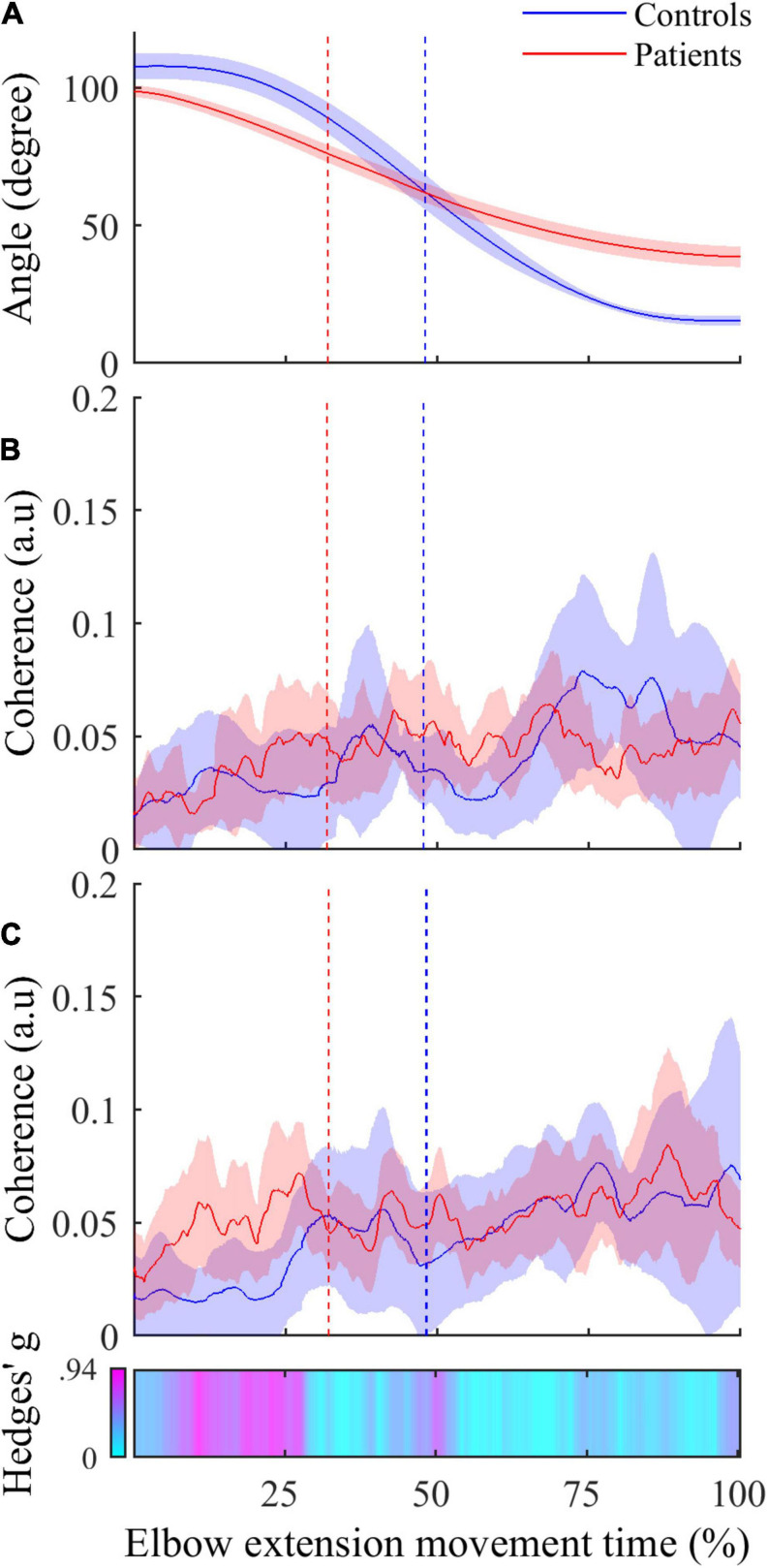
**(A)** Active elbow extension angle (mean ± 95% CI) as a function of time expressed in percent for controls (in blue) and patients (in red). **(B)** Mean instantaneous CMC with agonist TB muscle (mean ± 95% CI) as a function of time expressed in percent for controls (in blue) and patients (in red). **(C)** Instantaneous CMC with antagonist BA muscle (mean ± 95% CI) as a function of time expressed in percent for controls (in blue) and patients (in red). In **(C)**, CMC is higher in patients in more than half the acceleration phase and not different in deceleration phase when using non-corrected *p*-values (α = 0.05). Instantaneous Hedges’ g, representing effect size of the CMC difference are shown as a horizontal bar with values ranging from 0 (cyan) to magenta (0.94). In all graphs, the vertical dashed lines represent the boundary between the acceleration and the deceleration phases for controls (in blue) and patients (in red).

The comparison of instantaneous CMC to mean CMC values of each group showed a biphasic pattern for both healthy controls and post-stroke patients. However, CMC was significantly below the mean for the first 25% of movement duration for healthy controls (CMC < 0.02, 2.13 < *t* < 3.52, all corrected *p* < 0.05), while it was significantly below the mean during only the first 15% of movement duration for post-stroke patients (CMC < 0.035, 2.23 < *t* < 7.94, all corrected *p* < 0.05). Noteworthy, the same pattern for both groups was observed in the two other antagonist muscles (i.e., BB and BR), whereas it was not observed in the agonist muscle (i.e., TB) (results not shown).
• The inter-group comparisons of instantaneous CMC magnitude are shown in Figure 4. In reference to the inter-group differences of the amplitude of active elbow extension, point-wise comparison of the mean CMC with TB—i.e., with the agonist muscle—between the two groups did not reveal any difference (0.00 < CMC difference < 0.04, 0.01 < *t* < 2.17, all corrected *p* > 0.05). Similarly, the dynamic analysis of CMC with BA muscle did not reveal differences of CMC magnitude between the two groups (0.00 < CMC difference < 0.03, 0.01 < *t* < 2.43, all corrected *p* > 0.05). However, when compared to healthy controls, higher CMC was observed and subsequently confirmed by the effect size analysis (0.23 < Hedges’ g < 0.94) in post-stroke patients during the acceleration phase of elbow extension only. Noteworthy is that the same results were observed for the two other antagonist muscles (i.e., BB and BR) (results not shown).

## Discussion

This study originally investigated temporal dynamics of CMC in healthy subjects and post-stroke patients involved in a self-paced elbow extension movement to provide an understanding of whether the alteration of the functional coupling between brain and muscles contributes to motor function impairment. We analyzed kinematic data and changes of CMC magnitude over time during both the acceleration and deceleration phases of elbow extensions. Observed alterations of movement kinematics after stroke and changes in CMC compared to controls are discussed in relation to motor control theories in order to better understand the functional significance of CMC parameters in the neural motor control of agonist and antagonist muscles. The higher CMC levels observed in patients during the acceleration phase of the elbow extension is proposed to reflect the loss of selectivity of motor command occurring after stroke.

In line with the findings of previous studies (Murphy et al., 2011; Chalard et al., 2020), our results showed alteration of kinematic elbow extension performance parameters in post-stroke patients compared to healthy subjects. As expected, active elbow extension angle and extension angular velocity decreased in post-stroke patients. Besides these well-known motor function alterations (Parker et al., 1986), our results further confirmed that movement smoothness was also altered in post-stroke patients, but this group difference was specific to the deceleration phase of elbow extension. This finding complements previous results which have highlighted that stroke patients’ movement amplitude was related to movement smoothness (Murphy et al., 2011), and suggests an alteration of the central nervous mechanisms involved in the control of agonist or antagonist muscles during the braking phase of elbow extension. Indeed, even if it has been largely demonstrated that stretch reflex is increased in post-stroke patients (McPherson et al., 2018), i.e., a lower stretch sensitivity threshold leads to increased muscle contraction when the muscle is stretched, this hypothesis is supported by several key pieces of evidence. Among these evidence of increased antagonist muscle activity in post-stroke patients during voluntary movements, an association was found between altered beta cortical activity and excessive antagonist muscle activation (Chalard et al., 2020), and it has been shown that a less smooth movement is associated with recruitment of secondary motor areas of the brain during reaching and grasping after stroke (Buma et al., 2016). Referring to the single joint movement model proposed by Gottlieb et al. (1989) in the speed-insensitive condition which argues that only the duration of the excitation pulse to each muscle group is modified in a given task, herein we also saw evidence to suggest that the control of antagonist muscles may be less stationary after stroke compared to the situation in healthy subjects. We cannot exclude that agonist muscles weakening (Tang and Rymer, 1981) or inter-joint synergies impairment (Kisiel-Sajewicz et al., 2011) may take part in the alteration of movement smoothness. However, based on the assumption that agonist and antagonist muscles are controlled through the emission of continuous pulses triggering the agonists in acceleration phase and the antagonists in deceleration phase, we propose that post-stroke patients present sporadic excessive motor command to the antagonist muscles during the deceleration phase, which may at least partly contribute to altering the smoothness and, *in fine*, the performance of their movement (Chalard et al., 2020).

Both the average and instantaneous CMC analyses employed here clearly showed a significant amount of CMC during movement in all muscles for both healthy subjects and post-stroke patients. CMC is known to be highly dependent on experimental design (von Carlowitz-Ghori et al., 2014), task difficulty (McClelland et al., 2012) or computation method (Bigot et al., 2011), and the actual presence of CMC during movements is still controversial. While some studies showed a total disappearance of CMC during lever displacement (Kilner et al., 2000, 2003), others highlighted significant CMC during either ankle cyclical (Yoshida et al., 2017) or isokinetic arm movements (Liu et al., 2019). In light of this debate, our results lend support to the idea that the detection of significant CMC occurs during both isometric contraction tasks and dynamic movements. In the same way as the conclusions drawn from isometric contraction paradigms, the presence of CMC during dynamic movements could indicate that it reflects central mechanisms involvement in control of agonist and antagonist muscles. Besides the detection of significant CMC during the whole elbow extension movement, the analysis of the temporal dynamics of CMC revealed the presence of CMC magnitude variations during movement in antagonist muscles for both healthy and post-stroke subjects, suggesting non-constant coupling between cortical and muscular pools of neurons. Beyond the evident methodological interest of such an analysis of CMC, this finding reveals that the flow of afferent or efferent information reflected through CMC (Riddle and Baker, 2005; Witham et al., 2011) varies over movement duration. From a functional point of view, the presence of variable CMC during movement might thus reflect a movement-phase dependent sensorimotor integration participating in the online motor control along the visual control of trajectory (Elliott et al., 1999).

The intra-group analysis (see Figure 3) revealed that the temporal dynamics of CMC were different between controls and post-stroke patients in the acceleration phase. Compared to controls, patients showed a shorter period of non-significant CMC relatively to the total acceleration phase, suggesting an earlier functional coupling.

Even though the absence of significant instantaneous differences of CMC magnitude in the dynamic inter-group analysis may challenge this finding, some convincing evidence allow us to consider inter-group differences of CMC in antagonist muscles as meaningful differences of the functional coupling between brain and muscles. Firstly, the lack of significant differences can be related to the high variability of instantaneous CMC (see Figure 4). Given the massive number of observations in the dynamic analysis (> 2000), the correction for multiple comparisons, although mandatory, required less CMC variability and our analysis lacked statistical power allowing existing small differences to withstand the FDR correction. Secondly, by performing an instantaneous estimation of Hedges’ g (Hedges and Olkin, 2014) as a complementary analysis to evaluate the magnitude of the difference of CMC between both groups in antagonist muscles, we highlighted medium to large effect size reflecting meaningful differences (Cohen, 2013). Effect size analyses are thought to overcome the inherent limitations of the *p*-values and may reveal meaningful differences even if the statistical power of the study is relatively weak (Sullivan and Feinn, 2012). These complemental results are presented in the third panel of Figure 4 as a horizontal bar representing the instantaneous values of Hedges’ g in a cyan-magenta spectrum. According to the common interpretation of Cohen’s *d*-values (Cohen, 2013), the effect size of the difference in CMC magnitude ranges from medium to large effect (from 0.5 to 0.94) in roughly the same period highlighted in Figure 3 and does not exceed medium effect (< 0.5) during the remaining movement time.

In light of those converging arguments, the observed inter-group meaningful differences of CMC in antagonist muscles provide valuable additional evidence for the alteration of antagonist muscles control at the beginning of the movement in post-stroke patients. Indeed, these findings suggest an increase in the efferent and afferent information flow, which may be interpreted in different ways. Firstly, one may suggest that the presence of a premature CMC during the acceleration phase in post-stroke patients could be explained by the previously reported hypothesis from Witham et al. (2011): an earlier increase of afferent information flow in patients would be reflected in CMC quantification. Secondly, one may argue that the task of elbow extension does not involve the same motor control strategy in healthy subjects and post-stroke patients since patients may use compensatory strategies involving more than one joint while controls can easily perform the task with a single joint strategy. Even though the relationship between muscular synergy and CMC remains unclear in the literature (Reyes et al., 2017; Chen et al., 2018), the earlier emergence of CMC in post-stroke patients might reflect these different strategies. However, we rather explain the earlier detection of functional coupling between the cortex and the antagonist muscles during the acceleration phase in post-stroke patients by the fact that the central command to antagonist muscles could be altered during the acceleration phase with a concomitant trigger of both agonist and antagonist commands. Referring to the λ model of the equilibrium-point theory (Feldman, 1986), this interpretation appears consistent with the well-documented impairment of muscle selectivity in post-stroke patients, which has already been correlated to the loss of motor function (Lang and Schieber, 2004; Schieber et al., 2009). This model proposes that the central mechanisms are represented through two commands: the reciprocal command (R) which regulates the net torque production around the joint from agonist and antagonist activities and the coactivation command (C) which controls the simultaneous activity of agonist and antagonist muscles. In line with the study of Levin and Dimov (1997) on agonist/antagonist coactivation in healthy subjects and post-stroke subjects, the observed modulation of CMC may reflect that post-stroke patients present a modification of the temporal representation of the C-command, leading to an altered control of the concomitant activity of elbow extensor and flexor muscles and, *in fine*, an alteration of the co-contraction dynamics to the detriment of their motor function. Even though the activation of alternate motor fibers in patients (Rüber et al., 2012) could induce a concomitant noise, disturbing the synchronization through corticospinal pathways, we rather explain that the major mechanism underlying such alteration is the discrepancy observed in inhibitory mechanisms in post-stroke patients, both in the brain (Grefkes et al., 2008) and in the spinal cord (Katz and Pierrot-Deseilligny, 1982).

## Limits And Perspectives

One could point out few limits of this work. Firstly, a relatively small number of control subjects have been included, potentially reducing the probability of finding inter-group differences. However, the choice of strict statistical analyses reduced the possible biases and allowed for strongly supported discussion. The number of patients and the presence of antagonist co-contractions motivated the non-separation of the different patients according to their motor functions during movements even in patients with a good recovery level. Nevertheless, the individualized analysis of the association between the alteration of temporal patterns of CMC and the alteration of motor function would be interesting to support the hypothesis of the potential role of CMC as a marker of motor recovery (Krauth et al., 2019). Secondly, the EMG signals recordings and investigations could reflect a small degree of crosstalk between the EMG electrodes, mainly for antagonist muscles as BA and BR electrodes were located relatively close to each other. However, special care was taken to locate EMG sensors on the desired target muscle, and the potential impact of crosstalk between EMG electrodes can be regarded as low given that the intermuscular interactions were not studied in the present work. Nevertheless, further data collection should favor the use of intramuscular electrodes for an accurate measure of individual muscle activity. Thirdly, the high dynamicity of the task may have induced EEG contaminations from muscular contractions of the upper body. However, as was done in all previous studies by our group on EEG during upper limb contraction (Cremoux et al., 2013; Tisseyre et al., 2019; Chalard et al., 2020), each trial was visually inspected to remove trials where EEG signals were contaminated by muscle artifacts. Finally, even though the experimental task is the same for all participants, strategies to achieve the elbow extension may vary between subjects, especially for stroke patients. Given that our CMC magnitude comparison between healthy subjects and post-stroke patients might reveal different muscular synergies rather than actual alterations of the same central motor control mechanisms (Chen et al., 2018), the present results may require further investigations with more controlled dynamic tasks (and then less degrees of freedom for unexpected movement) to fully understand the part of the central motor control mechanisms supplied by CMC analyses.

The dynamic CMC analysis showed discontinuous levels of CMC magnitudes during elbow extension in both groups and suggested the presence of a fluctuating mix of afferent and efferent information taking part in voluntary movement control. The earlier functional coupling in the antagonist muscles during the acceleration phase of paretic elbow extension fits well to existing motor control models and to the contribution of the lack of motor selectivity to the loss of motor function in post-stroke patients. In line with recent neurophysiological studies (e.g., Coffey et al., 2021), our study supports the view that CMC, especially when considering its temporal dynamics for analysis, can be regarded as an appropriate tool for exploring the mechanisms underlying the online control of voluntary movements in healthy subjects and post-stroke patients, and can also provide a relevant ground for further analyses involving effective connectivity quantifications and dynamic causal modeling.

## Data Availability Statement

The raw data supporting the conclusions of this article will be made available by the authors, without undue reservation.

## Ethics Statement

The studies involving human participants were reviewed and approved by Research Ethics Board (No. ID-RCB: 2017-A01616-47) and Research Ethical Committee of Toulouse University Hospitals (No. 07-0716). The patients/participants provided their written informed consent to participate in this study.

## Author Contributions

MF co-wrote the manuscript, performed analyses, and discussed the results. AC and JT revised the manuscript, performed data acquisition, and discussed the results. DG and DA designed the study, co-wrote and revised the manuscript, and discussed the results. All authors contributed to the article and approved the submitted version.

## Conflict of Interest

The authors declare that the research was conducted in the absence of any commercial or financial relationships that could be construed as a potential conflict of interest.

## Publisher’s Note

All claims expressed in this article are solely those of the authors and do not necessarily represent those of their affiliated organizations, or those of the publisher, the editors and the reviewers. Any product that may be evaluated in this article, or claim that may be made by its manufacturer, is not guaranteed or endorsed by the publisher.
